# Medical and economic impacts of managing corneas from older donors at the tissue bank—a single-center retrospective study spanning over 12 years

**DOI:** 10.3389/fmed.2024.1415515

**Published:** 2024-10-24

**Authors:** Anne-Sophie Hatzfeld, Nicolas Germain, Patrice Maboudou, Mélanie Dhayer, Philippe Marchetti

**Affiliations:** ^1^Tissue Bank of Lille, Biology Pathology Center, CHU Lille, Lille, France; ^2^UMR 9020-U1277, CANTHER, University Lille, CNRS, Lille, France; ^3^Biochemistry Unit, Biology Pathology Center, CHU Lille, Lille, France

**Keywords:** cornea, tissue bank, donor, age, storage, endothelial cell density

## Abstract

**Aim:**

To evaluate the impact of corneas from donors over 80 years of age on the activity of the North of France Tissue Bank and to determine the potential cost implications for banks using corneas from older donors.

**Methods:**

We analyzed data from a single-center retrospective cohort study of 6,023 corneas preserved at the Lille Tissue Bank between 2012 and 2023. Donors, unrestricted by age, were divided into two groups: younger (≤ 80 years) and older (> 80 years). Corneas were categorized based on endothelial cell density (ECD). Data were collected from patients who underwent corneal transplantation. A financial impact model was created to assess the effects of using corneas from different age groups on the overall benefits of corneal transplant procedures.

**Results:**

The average donor age was 67.5 ± 14.5 years. The median age of donors gradually increased from 66 to 73 years over the 12-year study period, with donors over 80 years old representing more than 24% since 2021. Corneas from older donors had a higher discard rate (62.53% vs. 39.66%) due to poor endothelial quality and serological concerns (both *p* < 0.0001). Additionally, these corneas had lower ECD, with a larger proportion deemed unsuitable for grafting due to low ECD (30% vs. 8.2%). Corneas from younger donors were more often used for endothelial transplants, which require higher ECD. The mean economic benefit per cornea showed a moderate negative correlation with donor age. The net benefit of corneal transplants decreased as the proportion of donors aged over 80 years increased. It is predicted that a net benefit of zero would be attained when the proportion of donors over 80 years is 44.4%.

**Conclusion:**

Using corneas from donors over 80 years of age can help alleviate the shortage of donor tissue and be effective if certain quality standards are met. However, additional costs incurred by eye banks must be factored into this equation.

## 1 Introduction

Corneal transplantation is one of the most widely performed and effective transplantations in the world. The increase in the number of corneal transplantations over the past few decades can be attributed to several factors such as the growing number of aging patients who can benefit from grafts and advancements in surgical techniques. Considerable progress has been made in corneal transplantation. In addition to conventional penetrating keratoplasty (PK), endothelial keratoplasty (EK) procedures such as Descemet’s stripping automated endothelial keratoplasty (DSAEK) and Descemet’s membrane endothelial keratoplasty (DMEK) have gained popularity, enabling less invasive and more effective treatments for conditions such as Fuchs’ dystrophy and endothelial dysfunction. Microkeratome cutting is a precise surgical technique used in preparing DSAEK grafts, which are essential for certain corneal transplants. A microkeratome is a specialized tool with a very fine oscillating blade. In DSAEK, the microkeratome carefully slices a thin layer from a donor cornea, including the endothelial cells and a small portion of the stroma that will be transplanted. A major limitation of corneal transplantation is the varying supply of cornea available for grafting. Numerous countries face a shortage of donated corneas, particularly because the need for corneas is increasing at a faster rate than the number of corneas collected. The United Kingdom (UK) is estimated to face an annual shortfall of approximately 1,500 corneas (1).

Eye banks play a crucial role in connecting donors with recipients, ensuring that donated corneas are distributed to those in need, while adhering to the highest standards of quality, safety, and ethics (2). Eye banks evaluate potential donors for corneal transplant eligibility by conducting a thorough screening process that includes assessing medical history, administering risk assessments, and screening for infectious diseases (2). Additionally, eye banks assess the quality and suitability of the corneal tissue collected in an organoculture. One pivotal aspect of corneal assessment involves examining endothelial cell density (ECD) and morphology. The endothelium is critical for maintaining corneal clarity, and its viability directly affects the transplant success. Higher endothelial cell counts in donor corneas generally result in improved post-transplantation endothelial cell survival and graft longevity. Endothelial cell count is also an important factor in the allocation of donor corneas to various grafting surgical techniques, particularly when considering endothelial keratoplasty procedures, such as DSAEK and DMEK. Endothelial keratoplasty typically requires a higher endothelial cell count than PKP, because the transplantation process itself is traumatic to the cornea and results in a significant loss of endothelial cells. EK involves transplanting a thinner layer of tissue, which is more delicate and requires healthier endothelium for successful graft attachment and function (3). The quality of the endothelium can be influenced by several factors such as donor age, cause of death, postmortem time, and storage time (4).

In the 2000s, there was intense debate regarding the use of elderly donor corneas for transplantation (5, 6). Corneas from older donors have reduced tissue quality because of the natural aging process. The number of endothelial cells tends to decrease (7, 8) with age, potentially leading to corneal decompensation after transplantation, which explains the initial reluctance of surgeons to use corneas from older donors. The endothelial cell density (ECD) decreases by approximately 0.5% per year with age (9). The probability of ECD falling below 2,000 cell/mm2, the level at which the endothelium is unable to maintain normal function, increases in individuals over 70 years of age (10). This evidence has led to many eye banks worldwide setting age limits for corneal donors. Therefore, the upper age limit for corneal donation in Lille Tissue Bank was set at 80 years. However, transplanted corneas from elderly donors with ECD levels of –2,000–2,200 cells/mm^2^ have been shown to provide good visual outcomes, as this is the minimum required density for grafting (11). Furthermore, donors over 100 years of age can display viable endothelial cells suitable for grafting (12), suggesting that age alone does not necessarily indicate corneal quality. Thus, studies have concluded that age should not be a restriction for corneal grafting (13, 14). However, there is a potential advantage in using corneas from elderly donors. The use of corneas from older donors can expand the pool of available donor tissues, particularly considering that the lifespan of individuals in developed countries has increased. This can be especially beneficial during periods of donor corneal shortage. Therefore, in the early 2010s, eyebanks changed their selection process and extended the donor age limit to more than 80 years.

To the best of our knowledge, little data concerning the management of corneas from donors over 80 years of age have been published. For this reason, the objective of this retrospective study was to (i) evaluate the impact of corneas from donors over 80 years old on the activity of the North of France Tissue Bank and (ii) assess the financial impact on eye banks when utilizing corneas from older donors for corneal transplants.

## 2 Materials and methods

### 2.1 Corneal procurement and storage at the tissue bank

Between January 2012 and December 2023, the tissue bank of Lille preserved 6,023 corneas. The donors were not subject to age restrictions and the clinical selection process followed the recommendations of Agence de la Biomedicine. Serological tests were conducted in accordance with the European Directive.

Corneoscleral discs were obtained from 12 hospitals in northern France, using cadaveric or multi-organ donors. The collected corneas were stored in organ culture medium, either Corneaprep II (Eurobio, Les Ulis, France) or Stem alpha-1 from Stem Alpha (Saint Denis L’Argentière, France). Corneas were culture for 4–6 days at 31°C with periodic changes in the retrieval medium. Sterility was ensured by testing for bacterial and fungal contaminations. Before transplantation, the corneas were reassessed in a deswelling medium at either 31°C (Corneajet, Eurobio) or 20°C (Stem alpha-3, Stem alpha) and then transported to the graft site at room temperature.

Of the 6,023 corneas, 383 were preserved in serum-free Stem Alpha medium. Despite the differences in composition between the two media, a comparative analysis revealed no significant differences in endothelial cell density (ECD); the mean ECD for corneas preserved in Corneaprep was 2,446 cells/mm^2^, while for those preserved in Stem Alpha, it was 2,416 cells/mm^2^ (*p* = 0.2215) (data not shown). Thus, both media demonstrated equivalent performances in terms of ECD.

The study adhered to the principles of the Declaration of Helsinki and was approved by the ethics committee of the Lille Hospital. The following data were collected and analyzed: (i) donor characteristics (type of donation, age, sex, and virologic testing) and (ii) corneal characteristics (diameter, endothelial quality assessment, bacteriological and mycological results, and glucose and lactate concentrations in the organ culture storage medium).

### 2.2 Corneal process at the tissue bank

For endothelial quality assessment, the cornea was placed in a Petri dish with the endothelium facing upward and a drop of Balanced Salt Solution (BSS sterile irrigating solution, Alcon Lab, Fourth Worth, Texas, USA), which is a physiological irrigation solution isotonic to the tissues of the eye. A drop of trypan blue (Eurobio) was placed on the upper side to assess the endothelial cell mortality. The cornea was then rinsed with BSS and immersed in a 0.9% NaCl bath for three minutes to allow dilation of intercellular spaces. Subsequently, a drop of BSS was added to prevent drying of the cornea. The endothelium was then examined using a light microscope, and the endothelial cell density (ECD) was measured by counting cells using a grid system, where each square of the grid corresponded to a 0.01 mm^2^ area measured by a micrometric slide. The presence, number, and distribution of blue-stained cells; loss of cells; variations in cell size and shape; degree of folding of Descemet’s membrane; and diameter of the clear zone were evaluated. Finally, the corneoscleral disc diameters were measured. Donor corneas were evaluated by trained eye bank technicians and were systematically checked by a product manager. When the initial endothelial cell count determination is either impossible or yields equivocal results an intermediate control is conducted. This secondary assessment is critical in ensuring accurate and reliable data for the quality of the corneal endothelium, particularly in scenarios where the primary assessment is hindered by technical limitations or ambiguous findings. This process helps to mitigate potential risks associated with inaccurate initial readings and ensures that only high-quality corneal tissues are used in clinical applications. Based on these results, corneas were classified into three categories: unsuitable for grafting (ECD <2000 cells/mm^2^), suitable for PK (ECD > 2000 cells/mm^2^), and suitable for EK (DSAEK or DMEK) (ECD > 2,400 cells/mm^2^).

### 2.3 Glucose and lactate measurements

Glucose and lactate concentrations in the organ culture storage medium were measured at the end of storage using an ABL800 Flex instrument, and Glucose and Lactate concentration were measured in the organ culture storage medium using a SYNCHRON LX20 Clinical system (Beckman Coulter, Fullerton, CA, USA). Overall daily glucose consumption and lactate production were calculated as follows: (initial glucose − final glucose)/number days and (final lactate − initial lactate)/number days, respectively.

### 2.4 Recipient characteristics and transplantation outcomes

The corneal transplantation recipients (*n* = 827) in this study were all patients who visited one of the five local centers affiliated with the Lille University Hospital Tissue Bank. Patients who visited other centers (*n* = 798) were excluded from the analysis. The surgery type and indication were noted, and the frequency of each surgery, including regraft frequency, was calculated. The graft outcomes of corneas from donors were retrospectively analyzed.

### 2.5 Economic evaluation

Calculating the cost of a donor cornea involves several factors, including the cost of acquiring, processing, preserving, and distributing the corneas, which ranged from 395 € to 1,244 €. It is important to note that eye banks in France operate on a non-profit basis, and prices reflect an expense allowance rather than profit generation. Overhead costs associated with running the eyebank were excluded from this calculation. Revenues were determined based on the price at which each cornea was sold to hospitals or surgical centers, with prices ranging from 1,380 € to 1999 € depending on the type of cornea provided for PK or DSAEK. Naturally, the price of corneas has evolved over the 12-year period, accounting for rising costs. To assess the economic performance of the donor cornea bank, we calculated the expected net benefit or loss (revenues from suitable corneas minus the costs of all retrieved corneas) and adjusted it according to the donor age groups.

We developed a model to analyze the financial impact of utilizing corneas from donors of varying ages on overall profit from corneal transplantation. Specifically, we focused on distinguishing between corneas obtained from donors aged >80 years. The model is encapsulated by the equation *z* = 144.78 − 326*x*, where *z* represents the average profit in euros and *x* denotes the proportion of corneas sourced from donors over 80 years of age. This equation was derived under the assumption that corneas from donors younger than 80 years generate an average revenue of €144.78 each, while those from donors older than 80 years incur an average cost of €181.25 each, reflecting procurement and processing expenses. The model assumes a linear relationship between the proportion of older donor corneas used and overall profit, with profit decreasing as the proportion of corneas from older donors increases. This relationship is critical for optimizing financial outcomes in corneal transplant programs and provides a quantitative basis for strategic planning regarding donor-age demographics.

### 2.6 Statistical analysis

Statistics and graphs were produced using GraphPad Prism version 10.2.0 (GraphPad Software, La Jolla, California, USA). A two-tailed Fisher’s exact test was used to compare the characteristics of donors, corneas, and recipients. A two-tailed Mann–Whitney U test was used to identify differences in corneal glucose and lactate consumption, diameter, thickness, ECD, storage time, and recipient age. Pearson’s correlation coefficient was calculated and simple linear regression was performed to correlate ECD and Age. The Kaplan–Meier method with log-rank (Mantel-Cox) test and Gehan-Breslow-Wilcoxon test were used to assess corneal survival. Simple linear regression and correlation analysis using Spearman’s correlation were used to assess the correlation between donor age and the mean benefit per cornea. Statistical significance was set at *p* < 0.05; in charts, * *p* ≤ 0.05, ** *p* ≤ 0.01, *** *p* ≤ 0.001, and **** *p* ≤ 0.0001.

## 3 Results

**Figure 1:**
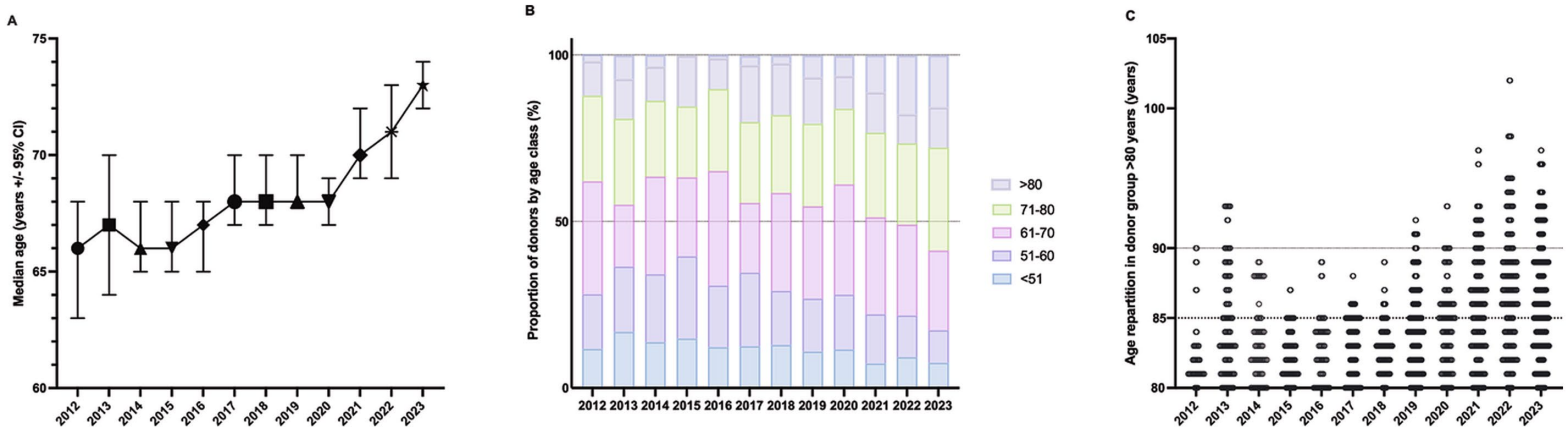
Age distribution of donors during 2012–2023. **(A)** Trends in the median age of corneal donors (*n* = 6,023) along with 95% Confidence Interval (CI) over a twelve-year period from 2012 to 2023. **(B)** Proportion of donors by age class (%) from 2012 to 2023. Total number of donors = 6,023. **(C)** Trend of age partitioning in donor groups over the age of 80 years (*n* = 1,245; each dot represents one individual). Secondary horizontal dotted lines at 85 and 90 years are shown.

The tissue bank of Lille received 6,229 corneas from Northern France between 2012 and 2023. Among the study population, the average donor age was 67.5 years, with a standard deviation of 14.5 years (median age: 69 years; 25^th^ –75^th^ percentile: 59–78 years). Most donors were aged between 61 and 70 years, while 20% were aged >80 years. A small percentage of donors were over 90 years old (2%). By examining the donor age distribution across years, we observed that the median age gradually increased from 66 years to 73 years over the 12-year study period. The data showed steady aging of the donor population, with the most significant increase occurring after 2020 (Figure 1A; Table 1). The most substantial augmentation was observed in the oldest age group (>80 years) (Figure 1B), representing more than 24% of donors since 2021, with donors over 90 years of age becoming more prevalent in later years (Table 1; Figure 1C).

**Table 1:** Donor age repartition.

Year	*n*	Median	25th	75th	Donors > 80 years (%)	Donors > 90 years (%)
2012	320	66	58	77	12.2	0.0
2013	435	67	56	78	19.8	1.8
2014	350	66	57	76	13.4	0.0
2015	405	66	55	77	15.8	0.0
2016	406	67	59	76	10.3	0.0
2017	460	68	56	80	19.4	0.0
2018	461	68	57	77	17.4	0.0
2019	745	68	60	79	20.7	1.1
2020	566	68	59	76	16.3	0.4
2021	600	70	62	80	24.7	5.9
2022	623	71	62	82	20.7	5.8
2023	858	73	65	82	22.3	5.6
Total	6,229	69	59	78	19.9	20.1

### 3.1 Baseline characteristics of donors older than 80 years

Of the 6,229 corneas, 1,250 (20.1%) were assigned to the older donor group (> 80 years) and 4,979 (79.9%) were assigned to the younger donor group (≤80 years). There was a significantly higher proportion of female donors and non-heart beating donor (NHBD)-type procurements in the older age group (>80 years) than in the younger age group (≤80 years) (Table 2, *p* < 0.0001). Moreover, Table 2 shows that, while the time from death to procurement was not affected by donor age, the suitability of the corneas for grafting was. The main reason for rejecting tissues of poor quality is the lack of suitable endothelial cell density for grafting. Corneas from older donors (>80 years) had a higher discard rate (62.53% vs. 39.66% in the younger age group), particularly because of poor endothelial quality (*p* < 0.0001) and serological concerns (*p* < 0.0001).

**Table 2:** Characteristics of donors according to donor age.

Donor characteristics	Donor age				
	≦80		>80		*p*
		*n*		*n*	
Mean Age ± (SD)	63.05 (12.66)	4,979	85.32 (3.675)	1,250	
Gender (%)					
Male	3,304 (66.05%)	5,002	518 (42.83%)	1,188	**<0.0001**
Female	1,698 (33.95%)		670 (57.17%)		
Type of procurement (%)					
MOHBD	1,245 (24.88%)	4,982	72 (5.74%)	1,247	**<0.0001**
NHBD	3,759 (75.12%)		1,182 (94.26%)		
Death to procurement time in hours ± (SD)	12.07 (6.676)	4,979	12.13 (5.887)	1,250	0.4723
Graft rate *n* (%)	3,002 (60.34%)	4,975	465 (37.47%)	1,241	**<0.0001**
Causes of discard					
Poor Endothelial quality	1,105 (22.21%)		519 (41.82%)		**<0.0001**
Invalid serology	369 (7.42%)		142 (11.44%)		**<0.0001**
Positive bacteriology	428 (8.6%)		94 (7.57%)		0.253
Invalid clinical selection	35 (0.70%)		14 (1.13%)		0.1493

Significant results are highlighted in bold to emphasize statistical relevance.

### 3.2 Characteristics of corneas from donors older than 80 years

**Figure 2:**
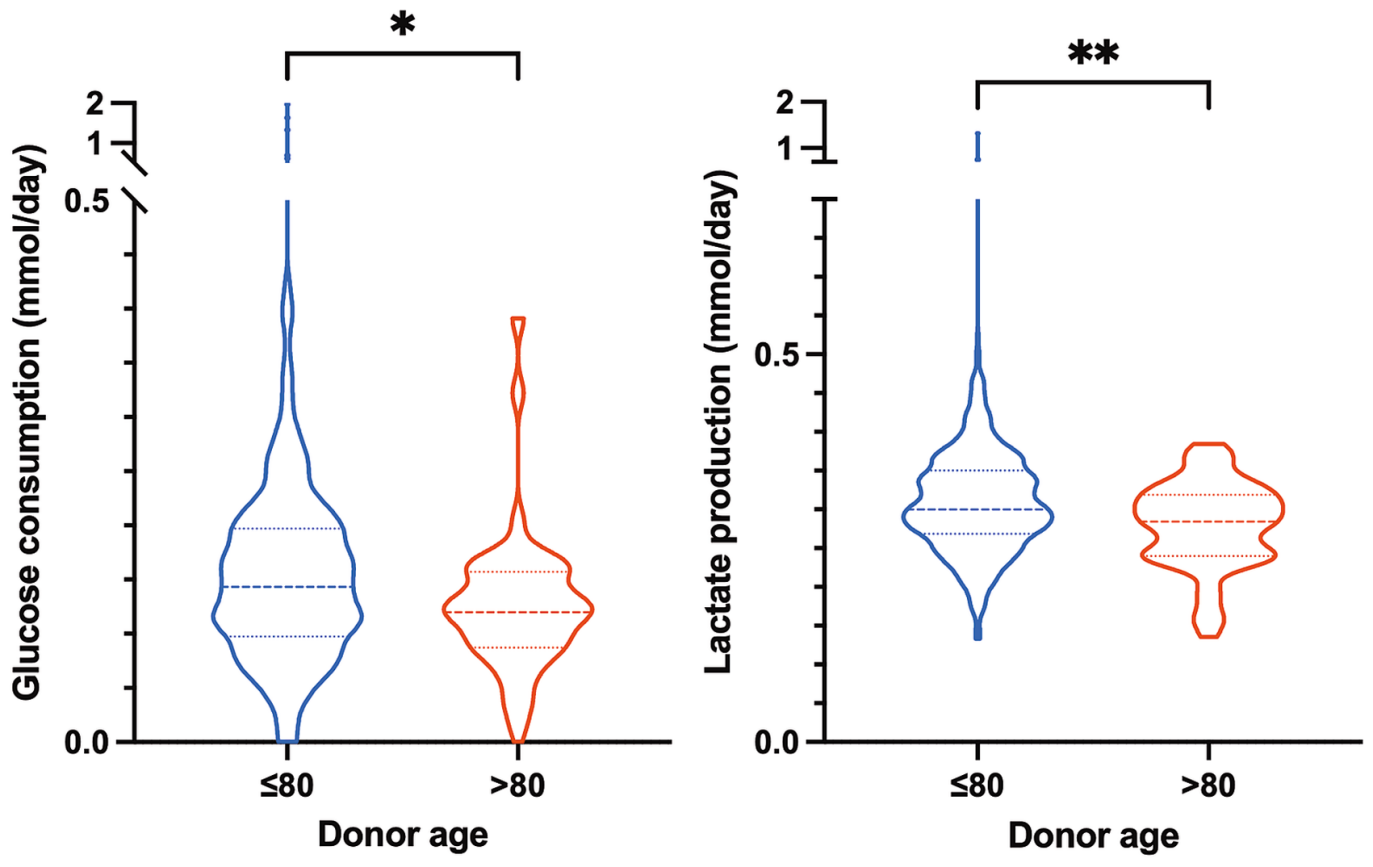
Comparative analysis of glucose consumption and lactate production in donor corneas across age groups. **(A)** Violin plot of glucose consumption (in mmol/day) of corneas from donors ≤80 years old (*n* = 389) and > 80 years old (*n* = 43). **(B)** Violin plot of lactate production (in mmol/day) of corneas from donors ≤80 years old (*n* = 606) and > 80 years old (*n* = 67). Mann–Whitney *p < 0.05, **p < 0.01.

Since functional corneas are glycolytic, we determined glucose consumption and lactate production in corneas according to age groups (Figure 2). Consistent with the poor endothelial quality, corneas from the older donor group consumed less glucose (mean, 0. 12 ± 0.07 nmol/day vs. 0.16 ± 0.16 nmol/day; *p* = 0.01) and produced less lactate (mean 0. 28 ± 0.05 nmol/day vs. 0.32 ± 0.08 nmol/day; *p* = 0.001) than those from the younger group (Figure 2). Next, we compared the corneal characteristics according to the donor age groups (Table 3). Corneas from donors older than 80 years had a significantly higher incidence of cataract scars (*p* < 0.0001), slightly smaller diameters (*p* = 0.0008), more corneas with corneo-scleral diameter > 15 mm that were unsuitable for DSAEK surgery (*p* = 0.0351), and lower endothelial cell density (*p* > 0.0001). Corneas from the >80 group were stored in organ culture for an average of 22.3 days, which was slightly longer than those from the ≤80 group, which averaged 21.8 days (*p* = 0.001) (Table 3), which could have contributed to the lower ECD (15). A greater percentage of corneas from younger donors required intermediate controls (14.98% vs. 8.08% in the older group; *p* < 0.0001).

**Table 3:** Characteristics of corneas according to donor age.

Corneal characteristics	Donor age				
	≦80		>80		*p*
		*n*		*n*	
Cataract scar (%)	294 (11.52%)	2,551	274 (34.77%)	788	**<0.0001**
Cornea diameter in mm ± (SD)	16.02 (1.492)	4,662	15.87 (1.480)	1,139	**0.0008**
Number of which < 15 mm (%)	733 (15.72)		209 (18.35)		**0.0351**
Thickness in mm ± (SD)	474 (168.8)	394	504.7 (85.94)	49	0.7485
ECD cells/mm^2^ ± (SD)	2,484 (417.9)	3,288	2,213 (530.1)	592	**<0.0001**
Cornea process in tissue bank					
Number of intermediate controls (%)	333 (14.98%)	2,223	63 (8.08%)	780	**<0.0001**
Duration of storage in days ± (SD)	21.82 (4.059)	2,715	22.37 (3.8)	434	**0.001**

Significant results are highlighted in bold to emphasize statistical relevance.

**Figure 3:**
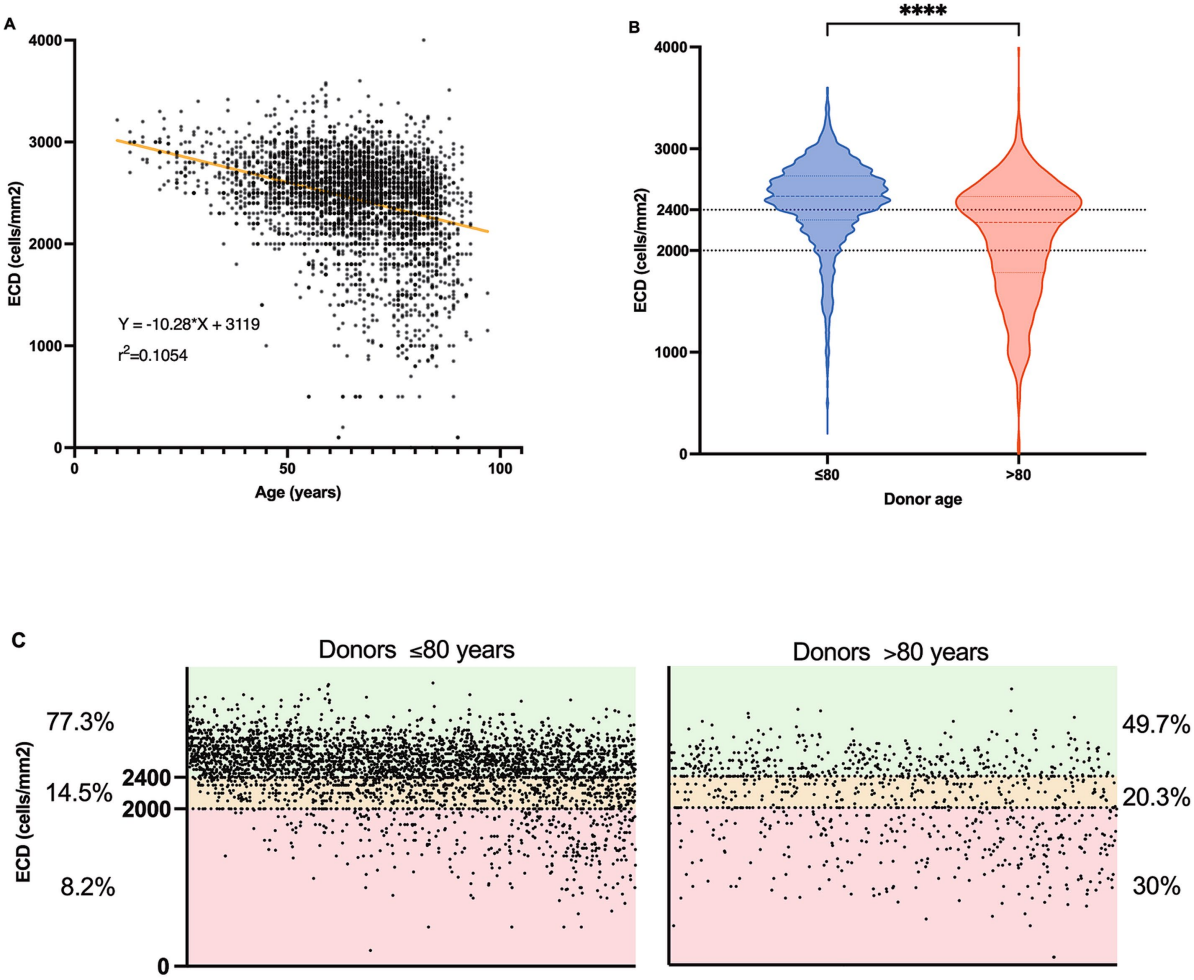
Age-related variations in Endothelial Cell Density (ECD) in corneas according to donor age. **(A)** Scatter plot depicting the relationship between donor age (*n* = 3,880) and ECD (cells/mm^2^). Simple linear regression line determined by the equation Y = −10.28X + 3,119 and a coefficient (r^2^ = 0.1054), *p* < 0.0001. **(B)** Violin plots to compare the distribution of ECD in cells/mm^2^ between two age groups of donors: ≤80 years (*n* = 3,936) and > 80 years (*n* = 908). Mann–Whitney ****p < 0.0001. **(C)** Scatter plots detailing the proportion of donors within specific ECD ranges for two age groups: ≤80 years (left part, *n* = 3,936) and > 80 years (right part, *n* = 908). Each chart categorizes ECD into three ranges (< 2,400 cells/mm_2_; 2000–2,400 cells/mm_2_, > 2000 *cf.* text for details), displaying the percentage of donors within each range.

We found a significant relationship between donor age and ECD (*p* < 0.0001) (Figure 3A). There was a significant difference in endothelial cell counts between younger and older donors, with older donors over 80 years having lower ECD on average (mean ECD 2129/− 553.7 vs. mean ECD 2479/− 408.3 for the younger group) (Figure 3B). We then ascertained whether there was a difference in the distribution of corneas suitable for grafting according to the donor age group (Figure 3C). For this, we distributed the corneas from donors of the two age groups into three categories according to their ECD [< 2000 cells/mm^2^: corneas considered unsuitable for graft; 2000–2,400 cells/mm^2^: corneas suitable for PK; > 2,400 cells/mm^2^: corneas suitable for either PK or endothelial keratoplasty (EK)]. In the associated graph, each point on the abscissa represents an individual cornea, allowing for the precise visualization and analysis of the data. Although there were still suitable corneas for grafting in the >80-year group, the proportion of those unsuitable for grafting due to low ECD (<2000 cells/mm^2^) was notably larger than that in the younger group (30% vs. 8%, chi-square *p* < 0.0001). Conversely, a higher proportion of corneas from the ≤80-year group was suitable for both types of keratoplasty procedures (PK and EK), with a particularly large number in the most preferred category (>2,400 cells/mm^2^) (77% vs. 49% in the older group).

### 3.3 Characteristics of recipients of corneas from donors older than 80 years

As the status of recipients can affect the success of the graft, we also compared the characteristics of the corneal recipients (Table 4). The age and sex of the corneal recipients were similar between the two donor groups. Concerning diagnosis leading to grafts, Fuchs’ dystrophy was more common in the ≤80 donor group compared to the >80 group (32% vs. 13%; *p* < 0.0001). Traumatic/infectious causes and graft decompensation were significantly more common in the >80 donor group (*p* < 0.0001 and *p* = 0.0232, respectively), suggesting that corneas from older donors were more likely to be grafted for traumatic/infectious causes or graft decompensation. This trend is likely influenced by the practice of matching donor and recipient ages where possible, given that older recipients are more likely to suffer from corneal pathologies such as trauma or infections that lead to graft failure. As the population of elderly recipients increases, they may be more frequently paired with older donor corneas, which could contribute to the higher incidence of complications observed in this demographic. Other diagnoses showed no significant differences in distribution between the two groups (Table 4). Corresponding to the diagnosis, endothelial keratoplasty was significantly more common in the ≤80 donor group (54%) than in the >80 donor group (34%) (*p* < 0.0001), whereas patch grafts were more frequent in the older donor group (6%) than in the ≤80 donor group (3.5%) (*p*-value = 0.0018).

**Table 4:** Characteristics of recipients according to donor age.

Recipient characteristics	Donor age				
	≦80		>80		*p*
		*n*		*n*	
Mean age (± SD)	65.82 (16.56)	710	66.85 (17.28)	115	0.2786
Gender (%)					
Male	338 (47.61%)		58 (50.43%)		0.6154
Female	372 (52.39%)		57 (49.57%)		
Diagnosis (%)					
Keratoconus	64 (9.01%)		10 (8.70%)		>0.9999
Fuchs’ dystrophy	229 (32.25%)		15 (13.04%)		**<0.0001**
Pseudophakic Keratopathy	175 (24.65%)		30 (26.09%)		0.7283
Traumatic/Infectious	63 (8.87%)		26 (22.61%)		**<0.0001**
Graft decompensation	114 (16.06%)		29 (25.22%)		**0.0232**
Keratopathy Bullosa	4 (0.56%)		0 (0%)		>0.9999
Stromal dystrophy	13 (1.83%)		3 (2.61%)		0.4784
Iterative grafts	23 (3.24%)		1 (0.87%)		0.233
Other	25 (3.52%)		1 (0.87%)		0.1587
Graft type (%)					
Transfixing Keratoplasty	278 (39.15%)		56 (48.70%)		0.065
Endothelial Keratoplasty	382 (53.80%)		39 (33.91%)		**<0.0001**
Deep Anterior Lamellar Keratoplasty	30 (8.12%)		9 (22.44%)		0.0982
Patch graft	20 (3.52%)		11 (5.77%)		**0.0018**

Significant results are highlighted in bold to emphasize statistical relevance.

### 3.4 Graft outcomes of corneas

**Figure 4:**
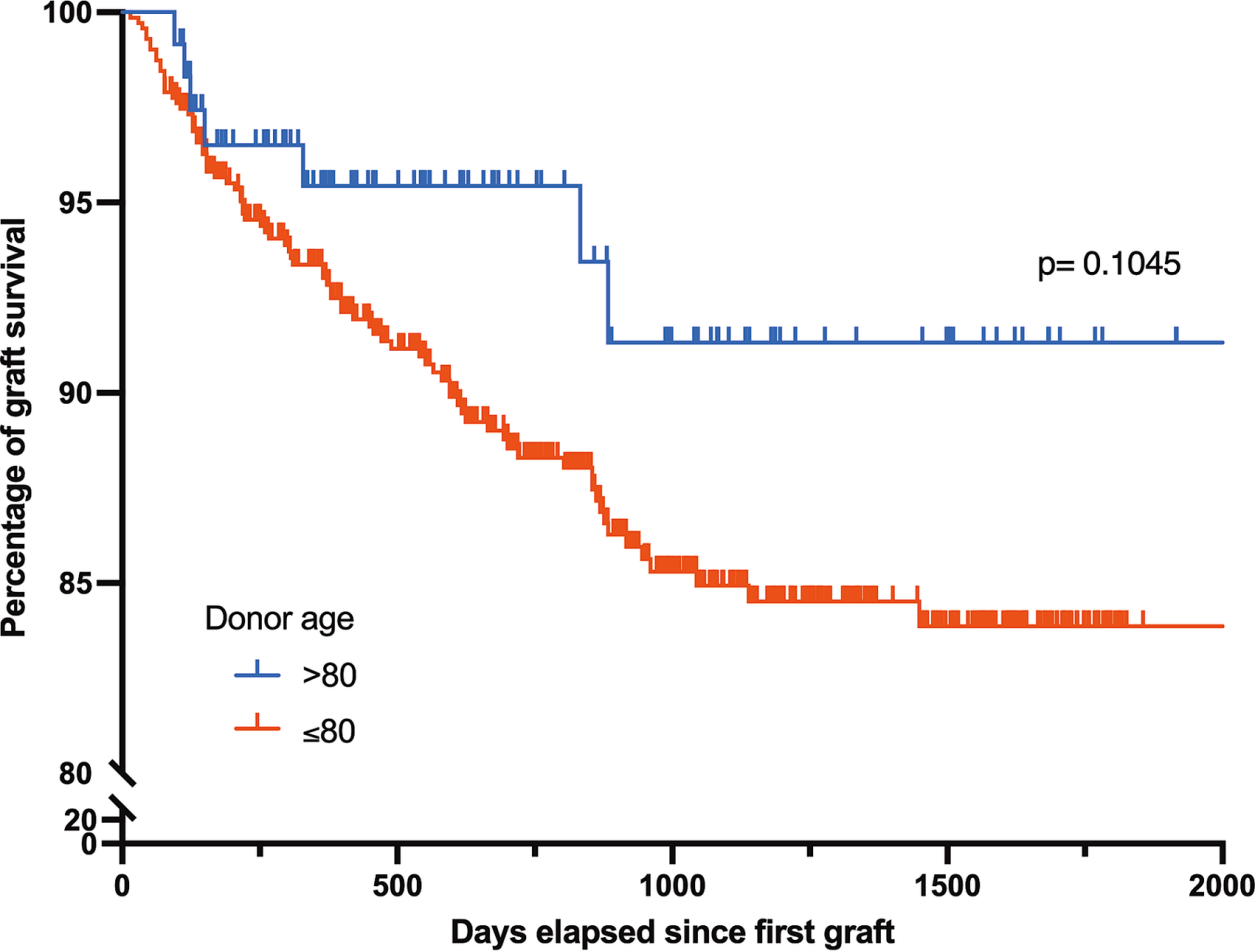
Longevity of corneal grafts by donor age group. Survival rate of corneal grafts as a function of time, comparing grafts from donors aged ≤80 years (*n* = 713) to those from donors aged >80 years (*n* = 119) over a period of up to 2000 days after the first graft; Gehan-Breslow-Wilcoxon test *p* = 0.1045, NS.

The overall regraft frequency was 10.8% (89/825), with no significant differences among donor age categories (Table 5). In the 89 regrafted patients, the mean time between the two corneal grafts was 385 ± 320 days. There was no statistically significant difference in graft survival times between older and younger donors (370 days ±388 in the >80-year group vs. 387 days ±320 in the younger group; *p* > 0.05). Similarly, when we compared the survival curves of corneal grafts (Figure 4), statistical tests showed no significant difference in graft survival between corneas from donors aged ≤80 years and those aged >80 years (*p* = 0.3038, Log-rank Mantel-Cox test). However, despite the lack of statistical difference in regraft rates between older and younger donors, there was a tendency for better survival of corneas from older donors. This may be explained by the fact that younger donors more frequently provide corneas for endothelial grafts, which are associated with higher regraft rates. Moreover, there was no significant difference in the regraft rates between the two donor age groups, regardless of keratoplasty type (Table 5). Overall, these results indicated that factors other than donor age may play a significant role in determining graft survival. However, it is important to note that the small sample sizes in some categories (especially in the >80 years group) may limit the power of statistical tests to detect differences.

**Table 5:** Analysis of the regraft rate as a function of donor age.

	Donor age				
	≦80		>80		*p*
		*n*		*n*	
Number of regrafts (%)	82 (11.55%)	710	7 (6.09%)	115	0.1034
Of which					
Transfixing Keratoplasty	24 (7.95%)	302	3 (5.08%)	59	0.5932
Endothelial Keratoplasty	50 (11.57%)	432	2 (4.88%)	41	0.2935
*DMEK*	5 (8.2%)	61	0 (0%)	2	>0.9999
*DSAEK*	45 (12.13%)	371	2 (5.13%)	39	0.2891
Deep Anterior Lamellar Keratoplasty	4 (11.76%)	34	0 (0%)	9	0.5636
Patch graft	4 (16.67%)	24	2 (15.38%)	13	>0.9999

### 3.5 Economic evaluation of corneal transplants at the tissue bank

**Figure 5:**
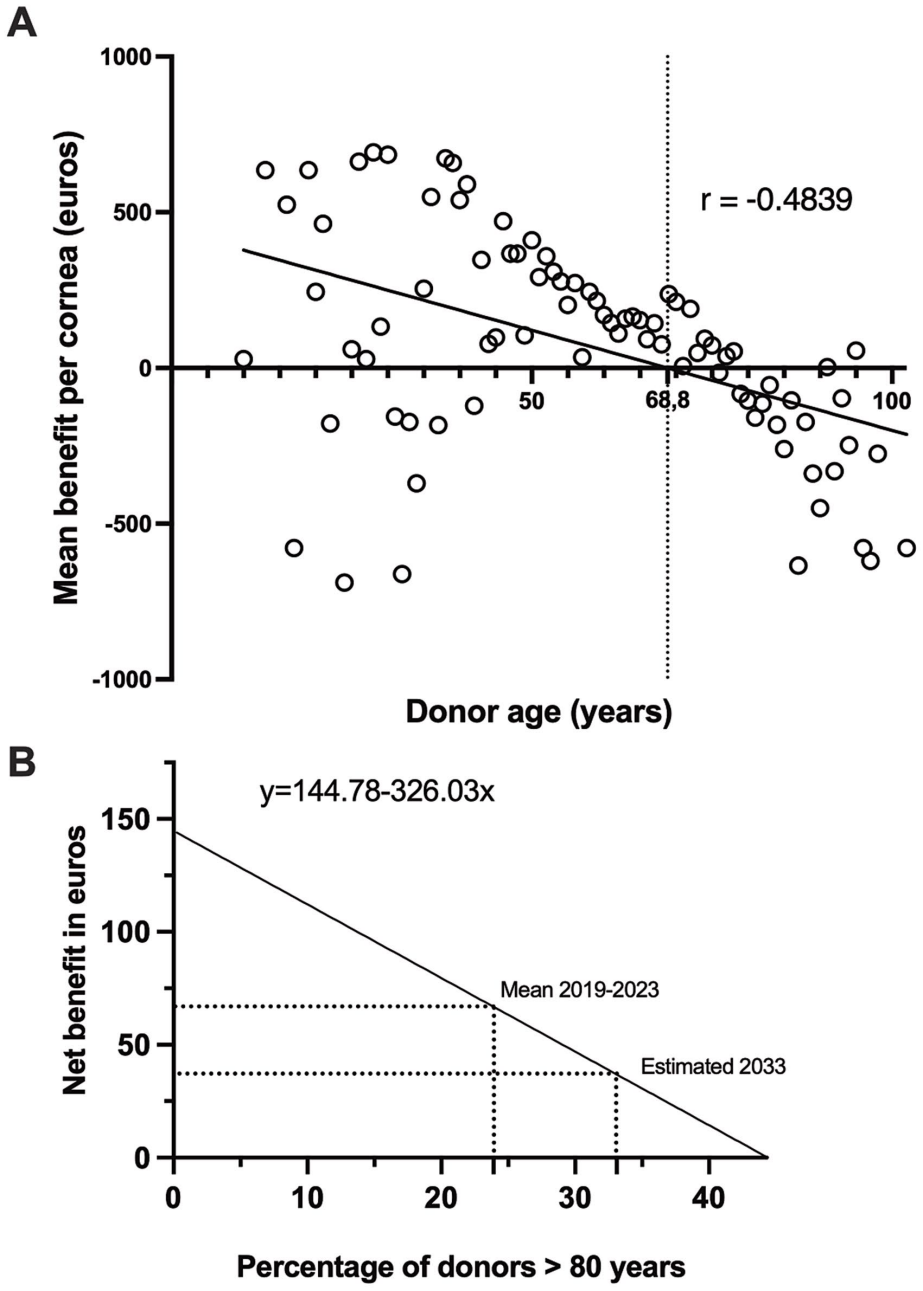
Impact of donor age and proportion of older donors on the economic benefits of corneal transplants. **(A)** Correlation between donor age (years) and mean benefit per cornea (in euros). Mean benefits estimated from to 2019–2023 data (*n* = 3,272, *p* < 0.0001). **(B)** Projected net benefit in euros against the percentage of donors over 80 years for the years 2019–2023 (Mean) and an estimation for 2033 (Estimated). The equation y = 144.78–326.03x illustrates the expected decrease in the net benefit with an increasing percentage of donors over 80 years.

Next, we performed an economic evaluation of corneal transplants categorized according to the donor age (Table 6). The mean profit per cornea for donors 80 years old or younger was positive. Conversely, for donors older than 80 years, the mean profit per cornea was negative, indicating an average loss for each transplanted cornea in this age group. There was a moderate negative correlation (*r* = −0.4839; *p* < 0.0001) between donor age and the mean economic benefit per cornea, indicating that older donor age was associated with lower economic benefits (Figure 5A). According to this correlation, the donor age at which the economic benefit shifts to a deficit is greater than 68.8 years. Figure 5B describes the net benefit in the euros of corneal transplants in relation to the proportion of donors older than 80 years, presented through a simple linear regression model. As anticipated, a negative correlation was observed between the percentage of donors aged >80 years and the net benefit derived from corneal transplantation, showing a decline in net benefit as the percentage of older donors increased. While the current data provide a model based on the past and present proportions of donors over 80 years, if the trend holds, the model can be used to make future predictions. This suggests that the net benefit will decrease further if the proportion of donors over 80 years of age increases, with a net benefit of zero when the percentage of donors over 80 years of age reaches 44%.

**Table 6:** Medicoeconomic analysis of the cost of corneas as a function of donor age.

	Number of corneas	Percentage	Cumulative expense €	Cumulative income	Cumulative benefit €	Mean benefit per cornea €
All donors	3,272		2308456.75	2,527,542 €	219085.25	66.96
Donor ≦80	2,491	76.1	1808035.88	2,168,680 €	360644.12	144.78
Donor >80	781	23.9	500420.87	358,862 €	−141558.90	−181.25

## 4 Discussion

The global shortage of donor corneas poses a significant challenge, with only one cornea available for every 70 needed based on the current demand (16). Globally, approximately 12.7 million patients are estimated to be in a queue for corneal transplant surgery. One way to mitigate this shortage is to maximize the use of collected tissues, including those from older donors, to meet the increasing demand for corneal transplantations.

Our observations revealed that the overall age distribution of corneal donors was over a 12-year period, with the most significant increase in age occurring recently. Previous studies have reported similar trends in aging of the donor corneal population (17, 18). The increase in the age of corneal donors could be attributed to improved health in older age, making older donors eligible for corneal donation, and changes in donation policies that now include older donors (19, 20). Additionally, it can reflect an increase in life expectancy in the general population, with older individuals choosing to donate corneas. Thus, collecting corneas from older donors can significantly increase the number of corneas available for grafting.

Our data also showed that the discard rate for corneas from older donors was significantly higher than that for corneas from younger donors, particularly because of the poor corneal quality and serological concerns. The latter finding is in agreement with the higher prevalence of positive serology in older donors (21). Clinical studies and eye bank data analyses have identified an inverse correlation between donor age and corneal endothelial cell density, which is a key determinant of the corneal quality (22, 23). Younger donor corneas are typically preferred because they tend to have higher endothelial cell counts and better overall quality (24). Donor age has been identified as a principal factor affecting the suitability and quality of corneas for procedures, such as PK and EK (22, 25). In this study, poor corneal quality was the main reason for rejection of corneas from older donors (Table 2). These include issues such as significant opacity, low endothelial cell count (usually <2,000 cells per mm^2^), and corneas affected by extensive scarring. Research has shown that corneal stiffness increases with age, with older corneas being stiffer than younger ones (26). This biomechanical property could make the cornea more difficult to sample and thus explain why we found slightly more small-diameter corneas in elderly subjects. Thus, we observed more aged corneas with a diameter of less than 15 mm, making corneas unsuitable for DSAEK.

Functionally cultured human corneas predominantly metabolize glucose (27). Here, we demonstrated that corneas from older donors consumed less glucose and produced less lactate than those from younger donors and that a reduction in glucose metabolism correlated with the age-dependent decline in corneal endothelial cell density. Several studies have reported a decrease in endothelial cell density over the course of a lifetime, which is consistent with the results of the current study [for review (28)]. However, it has been suggested that endothelial cells from older donors are more stable during organ culture than those from younger donors. When comparing corneas from donors aged <85 years with those from donors aged >85 years, we found that the former lost more cells during organ culture (13). This is further supported by the finding that cultured corneal endothelial cells from older donors displayed greater volume heterogeneity (29).

However, a moderate inverse correlation (*R*^2^ = 0.1054) implied that donor age alone is not a strong predictor of ECD. A wide range of factors influence corneal endothelial cell counts, including age, race, ocular pathology, surgery, and diabetes (30). Donor death also plays a significant role, with donors who die of long-lasting, severe diseases, such as cancer, with lower cell density (31).

The trend of poor-quality older donor corneas has medical implications as it may affect the suitability of donated corneas for transplantation. Several studies have found that corneas from donors aged >80 years are more likely to be discarded because of their poor endothelial quality (7, 32). In this study, > 60% of corneas from donors aged >80 years could not be used for transplantation (Table 2), which is consistent with the utilization rate of corneas from donors aged >75 years (33). Recipients from the ≤80 donor group were more likely to undergo endothelial keratoplasty, which could be related to the higher endothelial cell counts generally found in the corneas of younger donors (Table 4). This is primarily due to the lower proportion of corneas with an ECD greater than 2,400 cells/mm^2^ (Figure 3C), rendering older corneas less suitable for endothelial keratoplasties (DSAEK or DMEK). This is worrying in the context where endothelial keratoplasties have emerged as superior alternatives to PK for the treatment of endothelial diseases (34). These findings are important for corneal transplant programs, as they suggest that while older donors can contribute to the pool, their corneas are less likely to be used, and careful evaluation is necessary to determine their suitability. Interestingly, several innovative approaches are applicable to improve the survival and quality of corneas from older donors, including the use of Rho-kinase inhibitors (35).

Nevertheless, a significant number of corneas from older donors were grafted during the study. This also reflects the growing acceptance of surgeons for grafting corneas in older patients (36). This is justified because the use of corneas from older patients is associated with positive outcomes in terms of graft survival and visual rehabilitation (37). Consistent with recent studies (38, 39), we found that corneal grafts from donors aged >80 years had outcomes similar to those of younger donors. Successful corneal transplantation has been reported in patients over 90 years of age, with improved visual acuity and high graft survival probability (39). Similarly, the Cornea Donor Study (36) reported similar 5-year graft survival rates for corneas from older and younger donors, suggesting that age may not predominantly affect transplantation outcomes. Donor age does not appear to have an impact on the success rate of the graft after PK over a five-year period (40), nor does it seem to have an effect on postoperative endothelial cell loss and graft survival two years after DSAEK surgery (10). Additionally, there appears to be no discernible difference in visual acuity, endothelial cell density, and central corneal thickness between younger and older donor corneas 12 months after DMEK surgery when selecting corneas that meet the ECD criteria (9). These analyses indicate that donor age, in this case, may not be a decisive factor in the survival of corneal grafts (40). Studies have identified other recipient factors, as well as donor-recipient age or sex compatibility, as being more influential in rejection and post-transplantation results (41, 42). Age compatibility between the donor and recipient can influence the integration and functionality of the transplanted cornea, affecting both short- and long-term post-transplantation results (42). Certain conditions such as keratoconus have been associated with higher success rates than other conditions such as corneal trauma (43). These data highlight the complexity of the factors influencing graft survival, suggesting that donor age alone should not be the sole criterion for donor selection in corneal transplantation. Thus, while donor age may present challenges as long as the cornea meets the specific ECD criteria, it does not significantly affect the graft outcomes.

Additionally, it is important to note that, in France, all tissue banks follow strict policies and generally do not provide corneas from donors under 50 years of age for endothelial grafts, including DMEK. This decision was based on the fact that corneal lamellae from younger donors tend to curl more tightly, making them more difficult to manipulate during surgery, which increases the risk of complications and greater endothelial cell loss in the recipient eye. As a result, the use of corneas from older donors, provided they have good ECD, has become more common. Furthermore, while a high ECD is often sought, this may not always be justified, as research suggests that an ECD of 2,000 cells/mm^2^ is sufficient for 20 years of clear vision (44).

Our tissue bank adopted a cut-off of 2,400 cells/mm^2^ for endothelial grafts to compensate for the higher cell loss associated with these procedures. However, as the literature suggests that 2,000 cells/mm^2^ is sufficient for long-term graft survival, we may revisit our policy on endothelial cell density for endothelial keratoplasty in the future. This potential revision could allow for a more efficient use of available donor tissue, particularly from older donors, while still ensuring successful outcomes.

Economic evaluation of corneal transplants according to donor age is crucial for effective management of a tissue bank. We found that the mean expense for corneas from donors over 80 years of age was lower (Table 6) due to fewer intermediate controls (Table 3), as endothelial cell density (ECD) might be immediately unsuitable (less than 2,000 cells per mm^2^). Fewer necessary interventions owing to immediate disqualification could reduce the overall costs associated with these corneas. Furthermore, corneas deemed unsuitable for transplantation do not undergo a full array of tests or long-term preservation measures, which leads to cost savings. We then showed that corneas from donors aged 80 years and younger were more financially viable, with significant cumulative profit, whereas those from older donors resulted in cumulative loss, largely because of the higher discard rate (Table 2). Consequently, donors aged 80 years and below generated a mean net profit of 144.8 euros per cornea, while donors aged over 80 years had a mean net loss of 181.3 euros per cornea (Table 6). Therefore, our research demonstrated that on average, corneas from donors aged 80 years and younger yielded financial gains, whereas corneas from donors older than 80 years did not, highlighting the significance of donor age in the economic viability of corneal transplantation. This economic evaluation sheds light on the financial aspects of corneal transplants concerning donor age, and has significant implications for the financial management of corneal transplant programs. Based on the past and present proportions of donors over 80 years of age, we also created a model to predict the net benefit according to the percentage of older donors (Figure 5). This suggests an expectation for the net benefit to decrease further if the proportion of donors over 80 years increases by 44%, as the threshold percentage of donors over 80 years increases, at which point the net benefit becomes zero. This linear regression model can inform decision-makers about the upper limit of the proportion of older donors that can be economically accommodated.

However, this economic evaluation, focusing solely on banking aspects, does not necessarily reflect the clinical outcomes or quality of the transplants, and presents substantial limitations. The true cost of corneal transplantation includes presurgical evaluation, surgical costs, postsurgical follow-up, treatment of complications, and potential retransplantation. Additionally, economic assessments that do not consider surgical outcomes or the quality of life only partially evaluate the economic impact of the procedure. Decisions should balance financial considerations with clinical efficacy and ethical principles, to ensure that patient care remains the primary focus.

In summary, while using corneas from donors over 80 years of age can help alleviate shortages in donor tissue and may be successful if they fulfill quality criteria, they represent an extra cost for eye banks that could be considered, given the demographic evolution and the increase in the proportion of donors over 80 years of age in recent years. The decision to use corneas from older donors should be made carefully, considering the individual circumstances of the recipient and availability of suitable donor tissue.

## Data Availability

The raw data supporting the conclusions of this article will be made available by the authors, without undue reservation.
